# Case report: direct revascularization in acute mesenteric ischemia by an endovascular approach

**DOI:** 10.1186/s42155-019-0074-0

**Published:** 2019-09-03

**Authors:** P. Genzel, L. C. van Dijk, H. T. C. Veger, J. J. Wever, R. G. S. van Eps, R. M. E. Slangen, H. van Overhagen

**Affiliations:** HagaZiekenhuis, Els Borst-Eilersplein 275, 2545 Den Haag, AA Netherlands

**Keywords:** Acute mesenteric ischemia, Revascularization, Thrombosuction, Superior mesenteric artery

## Abstract

**Background:**

Acute mesenteric ischemia is a relatively rare but life-threatening clinical condition. Outcome depends on early diagnosis and prompt intervention.

**Case presentation:**

A 85-year-old man and a 75-year-old woman developed acute mesenteric ischemia due to cardiac embolism. The first patient received an insufficient dose of anticoagulants for atrial fibrillation and the second patient dicontinued her anticoagulantia to avoid bleeding during a routine colonoscopy. Both patients presented with severe abdominal pain and computed tomography showed thrombus in de superior mesenteric artery. Successfulrevascularization with good clinical outcome was achieved by means of an endovascular first approach.

**Conclusion:**

This case report shows that an endovascular approach - in contrast to open surgery - not only enables to revascularize main trunk lesions but can also facilitate revascularization of side branches. Endovascular treatment used to be limited to a selected group of patients without signs of bowel necrosis, but there is a tendency to initiate endovascular revascularization in all patients because it is associated with a reduced mortality, a reduced laparotomy rate and reduction in the resected length of bowel.

## Background

Acute mesenteric ischemia (AMI) is relatively rare, accounting for approximately one in 1000 hospital admissions in Europe (Kanasaki et al., 2018). This emergency caused by critically reduced intestinal blood flow requires early recognition and prompt intervention. Although AMI related mortality declined from 51% in 1995 to 26% in 2010, this is not associated with a higher revascularization rate, revascularization was only achieved in 6% of cases in 2010 in the United States (Kärkkäinen & Acosta, 2017).

Physical and imaging findings traditionally dictate therapeutic options in patients with AMI. Overt peritonitis or signs of bowel infarction or necrosis on computed tomography used to mandate open surgery. Prompt laparotomy allows for direct assessment of bowel viability and facilitates resection of necrotic bowel loops. However, a new trend in AMI is displayed in a recent guideline in this subject which recommends direct revascularization prior to resection (Björck et al., 2017).

These days, endovascular techniques due to technical improvements are increasingly used in several vascular territories including stroke and AMI. In open vascular surgery patients with AMI and main stem lesions of the superior mesenteric artery (SMA) can be revascularized by means of endarterectomy, but revascularization of distal side branches is technically demanding and often impossible. An endovascular approach, however, enables to revascularize both main stem lesion and also the side branches. This is illustrated in this report by two cases.

## Case 1

A 85-year-old man presented at our emergency department with acute severe abdominal pain, bloody diarrhea and dyspnea. Laboratory tests revealed leukocytosis (18,6 × 109/L), raised level of arterial lactate (4,5 mmol/L) and normal level of C-reactive protein.

Medical history included atrial fibrillation (AF), COPD Gold II and a TIA. AF was treated with Apixaban (Novel oral anticoagulant) 2.5 mg twice a day.

Contrast enhanced computed tomography in arterial and venous phases showed thrombus in the distal main trunk and branches of the SMA. There were no signs of bowel ischemia.

Percutaneous revascularization was attempted by a femoral approach. A 6 Fr sheath was introduced, followed by admission of 5000 IU of heparin.

The SMA was selectively catheterized with a 5 French cobra catheter from the groin and hereafter exchanged for a 6 French Guiding Sheath (Flexor Balkin Guiding sheath, Cook Medical, Bloomington, USA).

**Fig. 1:**
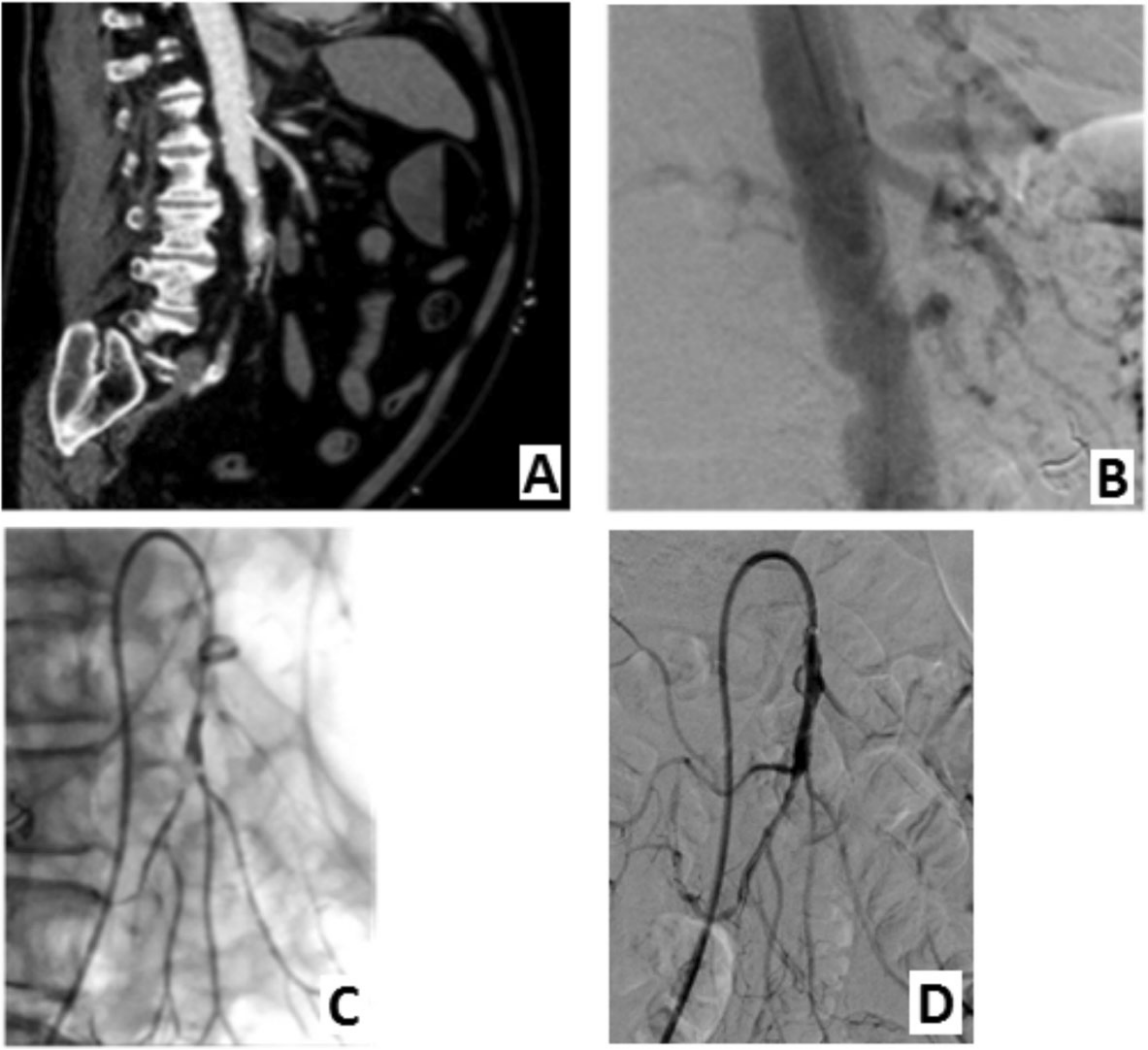
SMA embolism, occlusion main trunk and ileicolic artery (**a**) Sagittal CT image, arterial phase, main stem occlusion. **b** Lateral view DSA acute stop proximal in the SMA. **c** Selective SMA angiography after thrombosuction shows improved patency but residual thrombus in main stem SMA and ileocolic artery. **d** After PTA of ileocolic artery and main stem SMA followed by stent placement in main stem SMA shows patent SMA and side branches

Angiography confirmed a distal SMA trunk occlusion. Mechanical thrombectomy was performed using a Indigo System CAT6 aspiration catheter (Penumbra, Alameda, USA). This was followed by PTA with 3 mm balloon to improve inflow and pharmacologic thrombolysis by direct injection of 150.000 IU Urokinase. For the residual lesion in the distal SMA trunk a balloon expandable bare metal stent 8 × 24 mm (Palmaz Genesis, Cordis, a Johnson & Johnson company, New Brunswick, USA) was placed (Fig. 1).

The final angiogram showed successful recanalization of the SMA trunk and side branches.

The patient was admitted to the intensive care unit and received intravenous antibiotics and continuous i.v. infusion of unfractionated Heparin, target activated partial thromboplastin time (APTT) was set at 70–100 s.

A short period of relieve was followed by sudden deterioration characterized by excruciating abdominal pain within 24 h. Computed tomography angiography (CTA) showed mesenteric extravasation of vascular contrast material, indicating active bleeding. Laparoscopically no bleeding was visualized and there were no avital bowel segments. After discontinuation of heparin infusion there was spontaneous recovery. 16 days after initial revascularization this patient was discharged without bowel resection. He was subsequently treated with a therapeutic dosage of Apixiban (5 mg twice a day) because of AF.

## Case 2

A 75-year-old woman with a medical history of myocardial infarction and AF underwent colonoscopy after hemicolectomy for carcinoma several years previously. Her vitamin K antagonist medication was discontinued in order to avoid bleeding. Several hours after colonoscopy she developed abdominal pain and normal laboratory tests. CTA showed an occlusion of the SMA. There were no signs of bowel infarction.

**Fig. 2:**
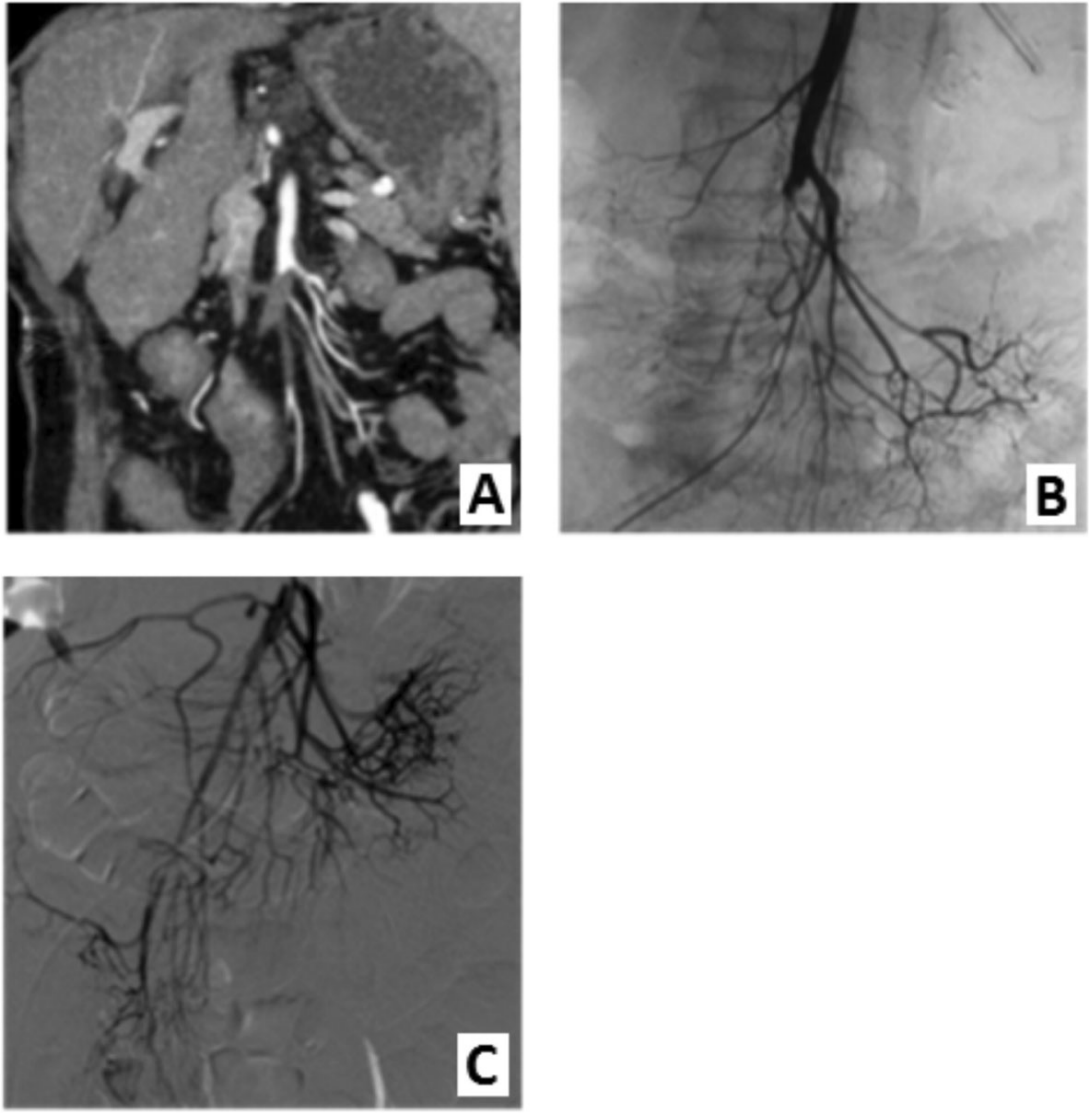
SMA embolism in proximal side branches. **a** CT image, coronal view, arterial phase. **b** An-giography showing disrupted blood supply particularly to the ileocolic artery and branches. **c** Restored blood supply in affected segments after thrombosuction, thrombolysis and PTA

She was admitted to the angio suite where angiography was performed through the groin. There was an occlusion of the SMA and several side branches. The SMA was catheterized and a 6 French Guiding Sheath (Flexor Balkin Guiding sheath, Cook Medical, Minneapolis, USA) was placed in to the SMA. The SMA was recanalized, thrombolysis was performed by infusion of Urokinase (250.000 IU) using a infusion cathter (Cragg-McNamara catheter, Medtronic, Minneapolis, USA) followed by thrombosuction and PTA with a 4 mm PTA balloon. The final angiogram showed a patent SMA and side branches (Fig. 2).

The patient underwent laparotomy in order to facilitate bowel inspection, there were no avital bowel loops. She improved clinically over the next days and was released from the hospital 14 days later.

## Discussion

These case reports demonstrate that modern endovascular techniques enable to revascularize not only the main stem of the SMA but also its side branches. Usually we catheterize the SMA with a 5 French Cobra or Celiac catheter and then exchange over a teflon 0.035″ wire for a 6 French guiding sheath, for example a hydrophylic Flexor Balkin (Cook Medical, Bloomington, USA). In case of a proximal stenosis or steep angulation of the main stem of the SMA a steerable sheath (TourGuide steerable sheath, Medtronic, Minneapolis, USA) can be chosen. Due to the adjustable tip angulation the acute angulation of the SMA is less challenging and a stable position can be reached.

Once a stable position with a guiding sheath has been obtained, mechanical thrombosuction (Indigo System CAT6 aspiration catheter Penumbra, Alameda, USA) for both main stem and sidebranches can be performed. If there is a residual lesion revascularization with a 0.014″ or a 0.018″ guidewire (e.g. glidewire Advantage (Terumo, Tokyo, Japan); V-18 (Boston Scientific, Marlborough, USA); Pilot 200 (Abbott Vascular, Santa Clara, USA)) followed by PTA with a 3 × 100 mm balloon in the side branches and a 5 × 40 mm balloon more proximal can be done. For remaining stenotic or occluded lesions in the main stem of the SMA stent placement is indicated, using either self-expandable stents(Carotid Wallstent, Boston Scientific, Marlborough, USA or SMART flex stent Cordis, Cordis, a Johnson & Johnson company, New Brunswick, USA), or ballloon- expandable stents (7 or 8 mm Palmaz Genesis, Cordis, a Johnson & Johnson company, New Brunswick, USA).

In case 1 we had to deal with a short remaining lesion in a quite large diameter vessel, therefore we chose a balloon expandable 8 × 24 mm stent.

Therapeutic options discussed here are applicable for AMI with thromboembolic occlusion of arteries, not caused by venous occlusion and non-occlusive mesenteric ischemia (NOMI).

There are no specific findings in physical examination or laboratory tests to confirm AMI. For decades the diagnostic process has been guided by imaging, which may facilitate a specific diagnosis but can also exclude other diseases. Non-contrast and biphasic (arterial and portal venous phase) contrast-material enhanced computed tomography with multi plane reconstructions has a high diagnostic sensitivity and specificity in AMI. CT findings in AMI include SMA occlusion, bowel wall thickening and decreased wall enhancement, bowel loop dilatation, mesenteric fat stranding and pneumatosis with or without portal gas. Diagnostic accuracy of imaging surpasses laboratory tests (D-dimer, Lactate and leukocytosis) and other diagnostic tools (Ginsburg, 2018; Kanasaki et al., 2018; Menke, 2010).

Until recently AMI and signs of non-viable bowel loops on CT were followed by laparotomy and bowel loop resection. Laparotomy may still be indicated but it is recently recommended that any bowel surgery should be preceded by revascularization (Ehlert, 2018). Revascularization can be performed by endovascular or open techniques. Open revascularization options include embolectomy with a balloon-tipped catheter through a distal transverse arteriotomy, and retrograde recanalization followed by antegrade stenting over a through and through wire (Acosta & Björck, 2014; Ehlert, 2018).

Endovascular approach (by brachial or groin access) should be preferred, given the possibilities to revascularize both the main trunk and side branches and the better reported results in the literature compared with open surgery.

Arthurs, in a retrospective single-center cohort study compared results of endovascular treatments vs. traditional operative therapy and reported a lower laparotomy rate (69% vs. 100%) and shorter length of resected bowel segments (52 cm vs. 160 cm) (Arthurs et al., 2011).

These trends are confirmed by Beaulieu et al., who reported on the National Inpatient Sample Database admissions from 2005 to 2009 and observed a lower mortality in endovascular vs. open treated patients (24.9 vs. 39.3%) and a lower bowel resection rate (14.4% vs. 33.4%) (Beaulieu, 2014).

Although these results indicate better clinical outcome after endovascular treatment, bias may have been introduced by selecting the clinical most fit patients for endovascular treatment and those with more advanced disease for open surgery.

## Conclusion

In patients with AMI new trends require endovascular first treatment when compared with open surgical revascularization and resection.

With an endovascular approach it may be possible to revascularize not only the main stem of the SMA with PTA and stenting but also to revascularize side branches by means of thrombosuction, thrombectomy and thrombolysis.

## Data Availability

Not applicable.

## Abbreviations

AF: Atrial fibrillation
AMI: Acute mesenteric ischemia
APTT: Activated partial thromboplastin time
CTA: Computed tomography angiography
IMA: Inferior mesenteric artery
NOMI: Non-occlusive mesenteric ischemia
SMA: Superior mesenteric artery
SMV: Superior mesenteric vein
